# Lecture Introductory to the Fifth Annual Course of Instruction in the Chicago Medical College, Medical Department of Lind University, Delivered Oct. 12, 1863

**Published:** 1863-11

**Authors:** N. S. Davis

**Affiliations:** Professor of Principles and Practice of Medicine, and of Clinical Medicine


ARTICLE XXXI.

### LECTURE INTRODUCTORY TO THE FIFTH ANNUAL COURSE OF INSTRUCTION IN THE CHICAGO MEDICAL COLLEGE, MEDICAL DEPARTMENT OF LIND UNIVERSITY, DELIVERED OCTOBER 12TH, 1863.

By N. S. DAVIS, M.D., Professor of Principles and Practice of Medicine, and of Clinical Medicine.

STUDENTS AND CITIZENS:—By the arrangements of the Faculty of this Institution, it is made my duty to open the present term of instruction with a formal address.

Just four years have elapsed since I had the pleasure of delivering the introductory lecture to the first course of instruction given in this College. As that occasion marked an era in the progress of medical education in this country, by introducing into the system of American medical college instruction a principle of great practical importance, I then deemed it proper to notice briefly the opinions and action of some of the most eminent members of our profession, in relation to the defects in the general system of medical education, and the paramount importance of correcting them; and as the completion of the new and commodious college edifice in which we are now assembled, marks an important step in the progress of the experiment inaugurated four years since, I shall offer no apology for recurring briefly to the same general topic that engaged our attention on that occasion.

It was then shown that the principles involved in the plan of organization and system of instruction adopted by the founders of this Institution, were neither new nor the invention of some eccentric or over-zealous medical reformer; but that they had been practically interwoven with the systems of medical education in every country on the Continent of Europe; and that they had been clearly pointed out and advocated by many of the ablest teachers and writers in America for more than twenty years past. Indeed, they are principles fully recognized and acted upon in this country, in all departments of education, except that which occurs in the medical schools. They are—

1st.—Such an arrangement of the several courses of lectures, comprising the annual college term, as will enable the student to restrict his attention, first, to the more elementary branches of medical science, and subsequently, to advance to the practical. Thereby making his medical college instruction progressive, as in all other institutions of learning, and not merely repetitional.

2d.—Such an increase in the number of professorships, and in the length of the annual college term, as will enable the faculty to include in each term a full review of all the important branches of medical science.

3d.—Such connection with hospitals as will make hospital clinical instruction an integral part of the regular college course, thereby making the instruction in the departments of practical medicine and surgery as fully demonstrative as in those of anatomy and chemistry.

For more than thirty years, the profession, through its representative men and through its State and National organizations, has urged such changes, in the organization and courses of instruction in the medical colleges, as would make them conform substantially to these propositions. Thus, the profession of Ohio, assembled in convention at Columbus, in January, 1838, discussed and adopted the three following resolutions, viz.:—

"*Resolved*, That, in the opinion of this Convention, the sessions of the different medical schools throughout this Union are too short, and that they ought to be extended one month, and the students required to stay to the end of the term.

"*Resolved*, That the number of professorships is too few, and that ampler provision be made for teaching physiology, pathological anatomy, pharmacy, medical jurisprudence, &c.

"*Resolved*, That, if practicable, our medical schools should be so organized as that students, in their first course, should have their attention chiefly directed upon special anatomy, physiology, chemistry, pharmacy, and other elementary branches; and, their second, upon pathological anatomy, therapeutics, the practice of physic, surgery, and obstetrics."

In the *Western Journal of Medical and Physical Sciences*, for March, 1838, we find the following comments on these resolutions, by the late Dr. DANIEL DRAKE, than whom no higher authority could be quoted on such a subject. He says: "Their first resolution, however, contains suggestions in which every reflecting member of the profession must concur. That the lecture terms in all the schools in the Union are too short, is undeniable. * * * * The second resolution furnishes a strong argument in support of the first, and ought, indeed, to have preceded it in the series. It looks directly to the limited range of studies prescribed and pursued in nearly every school in the Union. Indeed, we may affirm, that there is not one in which the cycle is as comprehensive as the nature of the medical profession demands." Referring to the third resolution, Dr. DRAKE continues: "It is not only absurd, but actually injurious, for the student who has recently commenced the study of medicine, and is not yet acquainted with the structure and functions of the body, with chemistry, or the rudiments of botany, or zoology, to engage the high and difficult inquiries of pathology and practical medicine; and, in the present organization of our schools, this is constantly done. The *beau ideal* of collegiate medical instruction would be for students, in their first course, to devote themselves to anatomy, special, general, and pathological, with dissections; to physiology, corporeal and mental; to chemistry, pharmacy, and the classification of medicines; and to so much of the history of the mineral, vegetable, and animal kingdoms as is necessary to the due understanding of the two last; and, in the second session, to give their chief attention to therapeutics, symptomatology, ætiology, practice, surgery, and obstetrics. * * * * * It is to be feared, however, that for a long time to come our brethren, who do not live in the immediate neighborhood of medical schools, will think, or at least act, differently from what is here advised; and, equally to be apprehended, that those who prescribe the policy of our institutions will neglect the establishment of junior and senior classes."

If the resolutions adopted by the Ohio State Medical Convention, and the comments of Dr. DRAKE, were just, as applied to the inadequacy of the schools, compared with the wants of the profession, twenty-five years ago, what shall we think of those schools that still adhere to the same annual college term of *sixteen weeks*, with six or seven professorships, while the actual field of investigation, requiring the careful attention of the student, has increased, at least, thirty per cent?

From the date of the Ohio State Medical Convention, to which we have alluded, to 1845, the subject of improving the system of medical college instruction in this country was frequently discussed in the several State Medical Societies. It was at the close of a protracted discussion of this subject, in the annual meeting of the New York State Medical Society, held in February, 1845, that a call was issued for a national convention of delegates from all the medical societies and colleges in the United States, for the special purpose of adopting measures in concert among all the schools. It was in response to this call, and mainly for the purpose of advancing the cause of medical education, that the American Medical Association was organized and has been vigorously sustained to the present time.

At the second annual meeting of that Association, held in Boston, May, 1849, the Standing Committee on Medical Education, of which Dr. F. CAMPBELL STEWART, of New York, was Chairman, made a lengthy and able report, in which the systems of medical education, both in Europe and America, were carefully reviewed. After thus reviewing the whole subject, and showing the great necessity of longer college terms, better preliminary education, and more system in the order of studies, the committee presented the following distinct recommendation, viz.:—"We think much might be gained by a division of the subjects taught into two classes; one series of which might be studied during the first course of lectures, and another during the second year's attendance. The anatomy and dissections, together with chemistry, materia medica, pharmacy, and physiology, might be studied during the first session; at the close of which, examinations should be held, and certificates of acquirement given. During the second session, the subjects of surgery, practice of medicine, midwifery, and hospital attendance, with a continuation of the study of anatomy, might be insisted on. This, we think, would be a decided improvement upon the present plan, which requires attendance on all the branches during both sessions, and does not permit the student time to prepare himself thoroughly on any one of them. We urge your close attention to this proposition, which we hold to be important, and which we think would be found to work well."

In a report made at the same meeting of the American Medical Association, by a committee appointed to consider, specially, the propriety of increasing the length of the annual college terms, we find the following explicit statement:—

"The plan of four month courses of lectures belongs to the origin of medical schools in this country, and arose out of the necessities of the case. The establishment of medical lectures at all was a bold innovation; and, lest it might act as a discouragement to students, the term was made as short as possible, and limited to four months. And yet, at that time, medicine had but a moderate expansion, and scarcely made pretension to a scientific character. Since the first establishment of the medical schools, the field of medical science has changed its entire aspect. The new departments that have been developed, exceed in extent, and equal in importance, the rudimentary branches forming the original scheme of medical education.

They embrace what may be correctly designated the higher and scientific branches of education. To include them with the original courses, in lectures of four month's duration, *is wholly impossible.*"

Similar sentiments have been expressed, and corresponding changes urged upon, the attention of the schools, by able committees, at almost every meeting of the Association up to the present time. But plain as are the principles of medical education already enunciated, and urgent and numerous as have been the appeals of the profession for their practical adoption by the schools, it was not until the organization of this Institution, in the autumn of 1859, that there was any attempt to carry them fully into effect in this country.

It is true, that many of the schools had so far yielded to the demands of the profession as to add two or three weeks to the length of their college terms, and one or two chairs to the number of their professorships. In two institutions, in which the professorships were endowed with permanent salaries, the annual terms were extended to six months or more, but the number of professorships was diminished rather than increased; and in none had there been an attempt to incorporate the all-important principle of arranging the various branches into junior and senior departments.

Under these circumstances, the Faculty of this Institution, some of whom had long been indentified with the efforts to improve the whole system of medical education in this country, determined to encounter all the dangers attendant on the abandonment of long-established customs, and at once deliberately enter upon the experiment of establishing a medical college, founded on sound educational principles and, in all respects, fully equal to the demands of the profession. They adopted a regular annual lecture term of five months, with the addition of a free summer reading and clinical term of four months. They increased the professorships to thirteen, dividing the branches taught into two series,—in such a way that the junior students could restrict their attention to the first or more elementary series, and the senior students to the more practical; thereby not only enabling the student to make his several courses of lectures progressive instead of repetitional, but also enabling him, during the whole period of his college attendance, to receive thorough instruction over a field of medical sciences twice as extensive as that covered in the ordinary college courses. They instituted daily examinations of the classes, and thorough general examinations of both junior and senior classes at the close of each lecture term. Hospital clinical instruction, both medical and surgical, was incorporated as a part of the regular college course. In a word, they boldly attempted to establish, practically and fully, what Dr. DRAKE had so happily described as "the *beau ideal* of collegiate medical instruction."

The enterprise was certainly not without obstacles and discouragements. An established medical college already existed in the city, with its alumni distributed throughout the whole North-West; and we were told that the patronage would not be sufficient to sustain two schools. Again, we were told that students could not be induced to attend a lecture term of five months, while another school in the same locality required an attendance of only sixteen weeks. It required only an accurate knowledge of the advantages of this city, as the natural centre of a vast territory, and including within itself, if properly developed, all the facilities for instruction in every department of medical science and art, to see that the first objection was entirely fallacious; and the force of the second could be tested only by actual experiment. Four years have now elapsed since this Institution, organized in the manner already indicated, began its career in rooms temporarily fitted up, not as facetiously remarked by an enemy of the enterprise, in the "loft of a warehouse," but on the third and fourth floors of an elegant block of buildings on Market street. The number of students attending the first annual lecture term was 33; the second 54; the third 63; and the fourth 81. Thus in the short period of four years, attracting a larger class than the old and justly celebrated medical departments of Yale or Dartmouth; and equal to the classes in one-fourth of the schools in the Union.

During the same period of time, by careful attention to the pecuniary income of the Institution, a museum has been filled with every needed means of illustration; a chemical laboratory, supplied with all the apparatus required in both departments of chemistry; and a library stored with more than one thousand valuable medical volumes. And, this evening, at the commencement of the fifth annual lecture term, instead of climbing three long flights of stairs to reach temporary lecture rooms, we are assembled in a new and permanent College edifice, admirably arranged for the work for which it was designed. On the first floor, is a library and dispensary room, a chemical laboratory, and the spacious lecture room in which we are now assembled. On the second floor, is a beautiful museum, and an anatomical and surgical amphitheatre. On the third floor, are the well-lighted and ventilated rooms for practical anatomy. All this we have, with a pecuniary encumbrance remaining of only six thousand dollars, payable in ten equal annual instalments.

Such, gentlemen, is a candid, though brief, statement of the origin, plan of organization, progress, and present condition of the CHICAGO MEDICAL COLLEGE, constituting the Medical Department of Lind University.

And here, while substantially dedicating this edifice to the great work of advancing the noblest of sciences; the science of alleviating human suffering and of prolonging human life; I take great pleasure in congratulating you, together with my colleagues in the Faculty, on the marked success of our enterprise thus far, and still more on the bright prospect that is opening upon the future. This pleasure is greatly enhanced by the fact, that our success has not been achieved at the expense of the medical school previously established in this city. On the contrary, her faculty has been stimulated to increased exertions, resulting in a corresponding increase in the number of students in attendance on their instructions. Hence the establishment of this Institution has already caused the aggregate number of medical students resorting to this city for college instruction to be more than doubled; while the hospitals and dispensaries, affording ample material for clinical instruction, have been enlarged and multiplied; and the means of illustration, in all the departments, have been greatly increased. Such has been, and such will ever continue to be, the effect of an enlightened and honorable competition. But, gentlemen, it is proper for me to remind you, that no medical college organization, however complete, can insure the development of accomplished and skilful physicians and surgeons, without the patient, well-directed, and earnest application of the student, during the whole period of his pupilage. The field of medical science, or more properly speaking, the field of sciences contributing directly or indirectly to a knowledge of the nature, causes, prevention, and cure of diseases is almost boundless. Some of its departments embrace the most intricate and intensely interesting problems in nature; requiring for their comprehension a mental discipline, a quickness of perception, and an intensity of application, which can be acquired only by close and protracted study. Permit me, then, to caution you against three errors, very prevalent among those persuing the study of medicine. The first is *haste*, or a desire to complete the period of pupilage in the shortest possible time.

*Haste* is said to be a national characteristic of Americans; and in no department of life is it exhibited more prominently than among medical students.

In Europe, the period of medical study required before becoming a candidate for graduation varies from four to seven years; while in this country, the rules of the profession and the laws of many of the States have fixed the period at three. And yet, short as this latter period is, when compared with the nature and extent of the studies required, there are many young men who manifest great anxiety to have it reduced still further; and not a few, who actually commence prescribing for the sick before they have read medical works eighteen months, or have gained even a superficial knowledge of anatomy, chemistry, and physiology.

Such haste to assume the practical duties and responsibilities of the profession, is not merely trifling with the highest interests of the sick, but it is a serious and permanent injury to the man who indulges it. In the first place, by commencing thus early to practice, his attention is necessarily absorbed in the efforts to procure formulæ or prescriptions for particular diseases, before he has any adequate knowledge of their pathology; or even of the *modus operandi* of the medicines he prescribes. Hence he speedily, and almost necessarily, glides directly into a purely empyrical or routine system of practice, without any other foundation than the *ipse dixit* of his preceptors and his books. Again, the failures and errors he almost necessarily commits while thus imperfectly educated, greatly retards the acquirement of that public confidence which is essential to professional success. Indeed, many are the cases in which the errors committed in premature efforts to practice, have cost half of the individual's subsequent professional career to outlive. As a mere matter of self-interest, therefore, no young man should allow himself to be hurried into the practical duties of the profession, by shortening the necessary period of study, or by passing hastily over the more fundamental branches of medical science.

The second mischievous error, committed by many of those who enter upon the study of medicine, is the neglect of mental discipline and of any adequate amount of preliminary education. Without having the mind trained to processes of reasoning, or habituated to the observation and comparison of facts and the logical deduction of conclusions therefrom, they enter, at once, upon the intricate and complex study of the structures and functions of the human system, with all the modifying influences of external agents on it. The living human body involves in its organization and functions almost every law or principle embraced in natural philosophy, chemistry, and physics; while a proper appreciation of the action of exterior agents on the animal economy, necessarily involves a knowledge of the facts of meteorology, geology, natural history, and physical geography; yet how large is the proportion of young men who commence their professional study, not only without the slightest knowledge of any of these branches of general science, but without even a respectable knowledge of English grammar and penmanship. We do not deny but that some of those, who have thus commenced, have ultimately attained a high degree of professional skill and an honorable position among their fellows. But they constitute the very few on whom nature has bestowed her choicest mental endowments; and who, consequently, would rise superior to all obstacles, however protracted and painful the task might be. Yet, even these few uniformly acknowledge that the deficiencies in their general education constituted a continual source of embarrassment, until actually supplied by eading and study, pursued at great disadvantage during hours snatched from the active duties of practice, and which should have been devoted to the cultivation of the higher and more intricate departments of medical science.

These same deficiencies, however, which constitute only impediments and annoyances to the progress of a few of the more highly gifted, stand as permanent and insuperable barriers to the progress of the many. With no independent knowledge of the natural and physical sciences, they can comprehend but imperfectly the application of the facts and laws of those sciences, in the elucidation of the causes of disease, and the nature and *modus operandi* of medicines; much less have they any basis on which to found new observations, or even to appreciate the value and bearing of such as may be forced upon their attention in the daily routine of life; and, if added to this, they have so limited a knowledge of language and literature, as to hinder them from freely expressing their views on paper, they must necessarily go through their whole professional career, as mere *prescribers* for the sick, neither satisfied with themselves, nor conscious of having made the world either wiser or better for their having lived in it. I know we are often told that all cannot become great or eminent in the profession. If by this it is meant that all cannot obtain official positions in connection with colleges and organized institutions, it is certainly true. If, however, there are not professorships or offices enough to accommodate the whole profession, it does not follow that each member may not attain a degree of professional skill and learning which would make him competent to fill any position. A

contemporary, while discussing this topic recently, comforted his audience with the assurance, that if only here and there one could attain eminence, the rest could remember that the *tall* oaks of the forest were the first to be riven by the lightning and the storm, while the lower and more obscure trees escaped unscathed.

Notwithstanding the abstract truthfulness of this figure, I think all will agree that a forest made up of stately oaks would be far more magnificent and valuable, than one chiefly composed of underbrush and saplings, with only here and there a majestic tree.

The third error, against which I wish to guard you on this occasion, consists in regarding the practice of medicine and surgery as a mere business calling, to be pursued like the various branches of commerce and the mechanic arts, simply for the pecuniary profits resulting therefrom. I do not here intimate that it is an error to receive pay for medical services. On the contrary, it is the duty of every practitioner to exact a fair compensation for his services, in all cases where the patient has the means to render it. But the error to which I allude, lies in a different direction. For instance, a very imperfectly trained mechanic may build a barn or a plain house, and receive compensation in proportion to the style of his work. Or an unskilful artisan may produce a fabric of very inferior quality, and sell it for a corresponding price; and no wrong is done to his patrons. So in all ordinary business pursuits, the varying conditions of men in society, require the exhibition of various degrees of skill with corresponding degrees of perfection in the products and results of labor. But the practice of medicine differs in two essential particulars from all other business pursuits. The first is, that every practitioner, whatever his qualifications may be, has to deal with the diseases and derangements of the same complex, intricate, and sensitive mechanism which we call the living human body. He cannot, like the mechanic, choose a subject to practice upon, whose organization is so simple as to be readily understood by a half-educated mind. On the contrary, whether educated or uneducated, skilful or unskilful, it is the same delicate, living, sensative organization with which he has to deal. Whether clothed in rags or in silks, it is the same human form divine; the last, most complex, and most beautiful of the Creator's works.

The second difference consists in the fact, that the results of the physician's labor affects not merely the pecuniary interests and conveniences of his patients, but their health, their happiness, and their lives. The merchant may sell us a fabric we do not like, but it can be laid aside or returned, and another procured in its stead. So an unskilful architect may plan a very inconvenient house, and another may correct his errors. But a lung disorganized through ignorance of the attending physician, or a limb sacrificed by an unskilful surgeon, cannot be restored or replaced, by the highest degree of human skill. Hence, from the very nature of the organization with which the physician has to do, it is evident that the practice of his profession, differs from all ordinary pursuits. And the all im-important truth, which I wish to impress indelibly upon your minds, here, at the very threshold of your professional career, is that you are under the highest moral obligations to so qualify yourselves, individually, as to afford every patient coming under your care, all the advantages which the present state of medical science and art is capable of affording. The question kept perpetually before your consciences, should be, not merely whether you have studied a certain number of years; or can pass an examination for a diploma; or are as well qualified as Dr. A. or Dr. B.; but whether you have so completely mastered all the facts and principles embodied in the various branches of medical science, and acquired such a degree of mental discipline as will enable you, promptly, to apply these facts and principles, with the highest attainable degree of skill, in the relief of human suffering?

Having thus explained to you the principles on which this Institution is organized, and the facilities it will afford you in the pursuit of medical knowledge; having cautioned you against undue haste in assuming the duties and responsibilities of practice; having pointed out to you the inconveniences and often fatal effects of neglecting a suitable preliminary education; and having placed clearly before you the practical standard of acquirements at which you should aim, it only remains for me to welcome you to the halls of this Institution; to its lecture rooms, its dissecting room, museum, library, dispensary, and the wards of the Hospital so closely allied to it. To all these, in behalf of the whole Faculty, I cordially welcome you; and freely pledge to you our most zealous efforts to facilitate your progress in the laborious and important work you have before you. In choosing the profession of medicine, you have, individually, assumed a high responsibility.

In your future lives, you will not only be in continuous conflict with disease and the grim monster, death, but you will be admitted to the inner circles of human society; to the fireside and the bedchamber of the rich and the poor; to the secrets of the vicious and the virtuous; to the sacred confidence alike of husband, mother, and daughter. Hence, it becomes you to sedulously cultivate all those qualities of head and heart, that characterize the true gentleman, the polished scholar, and the unswerving and incorruptible moralist.

If you faithfully and perseveringly heed these admonitions, you will not only attain a satisfactory and honorable position in the profession of your choice, and gladden the hearts of the afflicted at the sound of your footsteps, but you will be enabled to live in the enjoyment of the highest and purest degree of human happiness, the consciouness of having alleviated the sufferings and prolonged the lives of those around you.

